# Rare Cases

**Published:** 1871-09

**Authors:** Alfred L. Castleman

**Affiliations:** Milwaukee


### Correspondence.

### RARE CASES.

MILWAUKEE, *July 15, 1871.*

PROF. BYFORD,—DEAR SIR:—The liberty I, a comparative stranger, take in addressing you, will be excused, I hope, on the ground of the singularity of two cases which have lately occurred in my practice, and which it is the object of this letter to communicate.

On the 4th of March, last, Mrs. ——— aborted, and so suddenly that she had not time to get to bed before delivery of a fœtus of about four months. Her husband carried her to bed, and, in doing so, the fœtus, with cord, was detached at placenta. On examination, I found the os tincæ but little dilated, and so exceedingly sensitive that I deemed it best to leave placental expulsion to nature. After an hour or two, gave ergot—no effect; then uvi ursi, with no better success. After three or four days' confinement to bed she arose, and resumed her usual vocations, which she continued to follow with little or no inconvenience till the 21st of June, when I was summoned in haste to her bedside. Hemorrhage had suddenly occurred, and when I arrived she was cold and pulseless. I introduced two fingers and delivered the placenta, of normal size and *condition,* without the slightest approach to decomposition. The woman made rapid recovery.

Here is a case of placenta retained for nearly sixteen weeks, during the first four of which there was not even a lochial discharge; no enlargement of the breast, and for nearly four months the patient following her usual vocations—visiting her friends, and attending to household duties as usual. *At the end of four, and again of eight, and then of fourteen weeks, she menstruated regularly, and, to all appearance, healthily.* Has such a combination of symptoms and circumstances, usually, or even occasionally, been met with?

Two days ago, I visited a child three weeks old. At eight days old the nurse discovered milk discharging from the breasts; she daily, as she informs me, pressed from breasts one or two spoonsful of milk, and, as the secretion was increasing, the mother had the "milking" discontinued, with the intention of "drying up." When I saw her the mammary glands were hard, swollen, and inflamed, presenting in miniature a precise counterpart of an incipient abscess in breast of adult. I ordered poultices sprinkled with g. camphor. To-day, the inflammation is subsiding, and I hope to entirely arrest it without suppuration.

I shall deem it a great kindness on your part if you will inform me whether you have met with any such cases, or whether you are cognizant of similar recorded cases. I find none corresponding.

I am, dear sir, yours, very truly,

ALFRED L. CASTLEMAN, M.D.

